# Conservation implications of a mismatch between data availability and demographic impact

**DOI:** 10.1002/ece3.10269

**Published:** 2023-07-18

**Authors:** Alex Nicol‐Harper, C. Patrick Doncaster, Geoff M. Hilton, Kevin A. Wood, Thomas H. G. Ezard

**Affiliations:** ^1^ Ocean and Earth Science, National Oceanography Centre University of Southampton Southampton UK; ^2^ Wildfowl & Wetlands Trust Slimbridge UK; ^3^ Biological Sciences University of Southampton Southampton UK

**Keywords:** breeding propensity, common eider, matrix‐element elasticity, meta‐analysis, seaduck, vital rate

## Abstract

Cost‐effective use of limited conservation resources requires understanding which data most contribute to alleviating biodiversity declines. Interventions might reasonably prioritise life‐cycle transitions with the greatest influence on population dynamics, yet some contributing vital rates are particularly challenging to document. This risks managers making decisions without sufficient empirical coverage of the spatiotemporal variation experienced by the species. Here, we aimed to explore whether the number of studies contributing estimates for a given life‐stage transition aligns with that transition's demographic impact on population growth rate, *λ*. We parameterised a matrix population model using meta‐analysis of vital rates for the common eider (*Somateria mollissima*), an increasingly threatened yet comparatively data‐rich species of seaduck, for which some life stages are particularly problematic to study. Female common eiders exhibit intermittent breeding, with some established breeders skipping one or more years between breeding attempts. Our meta‐analysis yielded a breeding propensity of 0.72, which we incorporated into our model with a discrete and reversible ‘nonbreeder’ stage (to which surviving adults transition with a probability of 0.28). The transitions between breeding and nonbreeding states had twice the influence on *λ* than fertility (summed matrix‐element elasticities of 24% and 11%, respectively), whereas almost 15 times as many studies document components of fertility than breeding propensity (*n* = 103 and *n* = 7, respectively). The implications of such mismatches are complex because the motivations for feasible on‐the‐ground conservation actions may be different from what is needed to reduce uncertainty in population projections. Our workflow could form an early part of the toolkit informing future investment of finite resources, to avoid repeated disconnects between data needs and availability thwarting evidence‐led conservation.

## Graphical abstract

Demographic models supporting wildlife management depend on available information, making it imperative to prioritise collection of the right data. In the data‐rich exemplar of the common eider, breeding propensity is understudied relative to its importance. We present a workflow which could form part of a toolkit towards evidence‐led conservation.

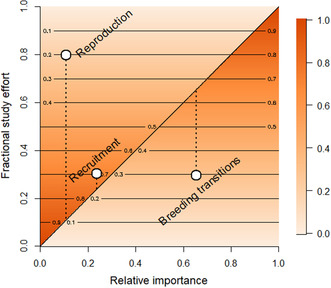

## 1 INTRODUCTION

In the face of population declines and species extinctions, biodiversity conservation functions as a crisis discipline (Díaz et al., 2019; Soulé, 1991). Limited resources compel conservation managers to triage their actions according to the best available data (Gerber, 2016). However, surveys of the state of conservation science have identified gaps in coverage and emphasised the important role of a ‘practice‐oriented research agenda’ in meeting the information needs of practitioners (Braunisch et al., 2012; Lawler et al., 2006). Targeted data collection is thus imperative, and population ecology plays a vital role in informing this process.

Mathematical population models are an essential component of the conservation toolkit (Frederiksen et al., 2014; Morris & Doak, 2002), but they often lack empirical estimates of the parameters needed for calibrating predictions. In a survey of mammals, birds, reptiles and amphibians, Conde et al. (2019) discovered a total absence of demographic data for just under 55% of the 32,144 species assessed, with a further 32% described only by summary measures. Stage‐specific survival and fertility values were available for <2%. Data deficiency thus inhibits biodiversity conservation because we lack foundational information across the life cycle on the probability of births and deaths, with quantitative information on the uncertainty around any estimates that we do have often insufficient (but see, e.g. Newton, 2010). Population modelling can circumvent data scarcity by directing research effort toward those vital rates that most influence projections of population dynamics (Heppell et al., 2000) or investment of conservation funds (Baxter et al., 2006).

Where they are accessible, vital rates stratified by stage (often age) inform our understanding of population dynamics (Caswell, 2001; Colchero et al., 2019). A common and accessible way to organise such rates is in matrix population models (MPMs), which represent (st)age‐structured life histories in a mathematical format that yields emergent properties with meaningful demographic interpretations (Caswell, 2001). A key contribution of MPMs to conservation biology is perturbation analysis, which identifies each matrix‐element contribution to the long‐term population growth rate *λ*, generating absolute sensitivities and relative elasticities (Caswell, 2001; Heppell et al., 2000). This is a prospective analysis considering theoretical future changes (i.e. if a vital rate were to increase by 10%, what would happen to *λ*?), in contrast to a retrospective analysis which quantifies past variability where long‐term empirical data are available (e.g. most variation in *λ* over the past 10 years was due to variation in vital rate A; see Caswell, 2000).

Hence, these measures present an opportunity to inform management options by identifying the most responsive stage for targeted intervention. For example, a classic study on a slow life‐history species, the loggerhead turtle *Caretta caretta*, found that while eggs and hatchlings received the majority of management interventions, *λ* was most influenced by juvenile survival. Egg protection alone would be insufficient to prevent eventual extinction, but population stability could be achieved with a 14% increase in juvenile survival, with turtle excluder devices suggested to reduce mortality from fisheries bycatch (Crouse et al., 1987). This provision of a candidate solution highlights the fact that influential vital rates (as identified by prospective analyses) will provide a useful conservation target, provided they are also amenable to intervention (likely those identified in retrospective analyses). For example, juvenile survival was found to have both the highest elasticity and ‘largest potential to be managed’ for Bonelli's eagle (*Aquila fasciata*) in a study by Soutullo et al. (2008, p. 1018), reminiscent of the ‘scope for management’ analysis formalised by Norris and McCulloch (2003).

The choice of vital rates for empirical study is often decided by other priorities than their influence on demography. For instance, while adult survival will invariably be considered wherever data availability allows, breeding propensity, which describes the probability of established breeders attempting breeding in a given year, is less commonly estimated in the field, due in part to the frequent assumption that individuals will continue to attempt breeding every year after recruitment.

When breeding propensity is estimated and found to be <1, it is often incorporated into MPMs simplistically, through a proportionally reduced fertility. For example, if breeding propensity were 0.75—that is, only three‐quarters of individuals attempt breeding in any one season—fertility would be reduced by one‐quarter (e.g. Etterson et al., 2011, equation (1b)). However, breeding propensity can be modelled more flexibly by distinguishing breeding and nonbreeding states, through incorporation into the transitions (e.g. Fujiwara & Caswell, 2001). This formulation provides scope to add model complexity but also biological realism based on the distinct underlying mechanisms of attempting to breed (as measured by breeding propensity) and subsequently raising offspring (fertility).

The choice of breeding propensity model is not merely a technical consideration. Longitudinal studies from across the animal kingdom have revealed prevalent intermittent breeding, whereby some established breeders skip one or more years between breeding attempts (e.g. Desprez et al., 2018; Rivalan et al., 2005). One such case is the common eider (*Somateria mollissima*), a widespread and abundant species of seaduck, with a long lifespan, deferred breeding and iteroparous life history. This much‐studied species presents a relatively data‐rich exemplar of a slow life‐history strategy (Koons et al., 2014). Despite its abundance, the IUCN Red List categorises the common eider as ‘Near Threatened’ globally and ‘Endangered’ in Europe, where it is projected to decline by 63% over three generations to 2033 (BirdLife International, 2018, 2021). Eider conservation managers thus have much to gain from understanding which of its life‐history components contribute most to population change and whether data collection efforts have reached data sufficiency.

In order to assess whether life‐stage transitions have been studied in approximate proportion to their influence on population dynamics, we first conducted a meta‐analysis of vital rate estimates, from literature review and a call for unpublished data (Nicol‐Harper et al., 2021a). Our meta‐analytic estimates then allowed us to parameterise an MPM with weighted mean values to investigate which life‐stage transitions most influence common eider population projections. Finally, we compared these matrix‐element elasticities to their respective data availabilities, assessing our findings against the likelihoods of mismatch predicted by probability analysis. We repeated our methodology to perform reanalyses of published studies. Our workflow should therefore inform future data collection for other species, towards more resource‐efficient and informative population modelling and management.

## 2 MATERIALS AND METHODS

### 2.1 Data synthesis

#### 2.1.1 Data collection

This study uses the vital rate database from Nicol‐Harper et al. (2021b), for which data gathering was supplemented with a call for data through the IUCN Species Survival Commission's Duck Specialist Group. We consider this to be equivalent in purpose to requesting information from agencies with a management mandate, as advocated for meta‐analyses by Beston (2011). Here, we use the same vital rates as in the linked database: first‐year survival (alternatively measured from hatching or fledging); second‐year survival; adult annual survival; recruitment propensities; breeding propensity of established female breeders; clutch size; hatching success; and fledging success. We facilitated screening of studies and estimates by assessing verification status (whether we found the estimate in its original source, or only a citation thereof); precision (whether the estimate presented was a point estimate or simply the midpoint of an observed range); and independence (in relation to multiple estimates provided by the same study, or separate studies based on the same datasets). Verification and precision were subject to sensitivity analyses (see below and Appendix A), while decisions and results relating to independence are described below and illustrated with an example in Appendix B. We focus on female–female transition rates for analytical tractability (Caswell, 2001).

#### 2.1.2 Meta‐analysis

As suggested by Earl and Fuhlendorf (2016), we did not apply formal meta‐analysis to vital rates with fewer than 20 independent estimates, which also lacked sample sizes for more than one‐third of the independent estimates; instead, for these we calculated simple means. For the vital rates undergoing formal meta‐analysis, we first checked whether means were sensitive to inclusion of unverified estimates or estimates based on range midpoints. Such sensitivity analysis, as recommended by Lajeunesse (2010) for cases where selected subsets of data may lead to different ‘pooled conclusions’, revealed that inclusion of unverified estimates, or those based on range midpoints, did not significantly alter mean estimates (see Appendix A). We therefore retained these estimates for completeness, in line with Beston's (2011) meta‐analytic approach to include third‐party data where original sources are unavailable due to language or access restrictions.

Where a study presented multiple estimates for one vital rate, we either (i) conducted a sub‐meta‐analysis across spatiotemporal replicates within a study (following a similar protocol to our main meta‐analyses), or, if this was inappropriate or not possible, (ii) selected the most appropriate for our purpose (e.g. the most precise, with justification provided in the database metadata). Our use of the term ‘sub‐meta‐analysis’ differs from that of Zoogman et al. (2015), but our decisions align with the suggestions of Mengersen et al. (2013 [Situation 1 in table 16.1]) and Haddaway et al. (2020 [Problem 7 – mitigation]) for maintaining independence among heterogeneous samples. In all cases, the aim was to ensure that meta‐estimates were calculated on independent replicate observations. All inclusion decisions are described within the database metadata and/or our provided code.

Variance estimates were included when available or calculable, to inform precision‐weighting (see below and Borenstein et al., 2009). For survival estimates based on mark–recapture modelling—such as using Program MARK (White & Burnham, 1999)—the standard error outputted from the programme was squared to obtain the variance for the populations from which the sample was drawn.

Our meta‐analyses used a random effects error structure, to allow for likely variation in population means across the geographic range of the common eider (Frederiksen et al., 2005; Guéry et al., 2017). We followed protocols in Doncaster and Spake (2018) for mean‐adjusted precision‐weighting, which removes a bias in meta‐estimation caused by inclusion of studies with little replication. This method also allows precision‐weighting of studies that provide replication but no variance estimate, on the assumption that the average of available variances applies to all studies (see Doncaster & Spake, 2018). The mean adjustment uses *s* ^2^, the mean of study‐level variances $s_{i}^{2}$, to calculate an error variance for each study *i*:

$$v_{i}=s^{2}/n_{i},$$

where *n*_*i*_ is the sample size of study *i*. The study‐level error variance *v*_*i*_ informs the precision‐weighting of each study‐level mean *δ*_*i*_, with lower *v*_*i*_ expressing higher precision. For a random effect, the appropriate precision‐weighting is:

$$W_{i}=1/\left(v_{i}+T^{2}\right),$$

where *T* ^2^ is the estimate of between‐study variance. For a one‐sample mean, we obtain an unbiased estimate of *T* ^2^ from Cochran's τ^2^ estimator (see ‘Hedges and Olkin method’ in Veroniki et al., 2016):

$$T^{2}=\mathrm{var}\left(\delta_{i}\right)–\text{mean}\left(v_{i}\right).$$

Finally, the standard one‐sample meta‐estimation by the mean of weighted means equals:

$$\left(\Sigma \;W_{i}\;\delta_{i}\right)/\Sigma \;W_{i},$$

with associated meta‐variance equal to 1/Σ *W*_*i*_. Studies thereby contribute to the meta‐estimate and meta‐variance in unbiased proportion to their precision (see Doncaster & Spake, 2018). Appendix B shows a worked example across a subset of the adult survival dataset.

### 2.2 Modelling

#### 2.2.1 Life‐cycle formulation and matrix population model

Seaducks are modelled as birth‐pulse populations, because they have a defined breeding season within the annual cycle (Caswell, 2001; Morris & Doak, 2002). Population projections must consequently choose to start the annual cycle either pre or postbreeding. We used a prebreeding life cycle, in recognition of the complications that can arise from postbreeding formulations (Kendall et al., 2019). The life‐cycle diagram and MPM thus project individuals from just before laying in year *t* to just before laying in year *t* + 1. This means that the youngest individuals at the start of the time‐step are just less than a full year old, referred to as 1‐year‐olds. Following projection, they will be just short of 2 years old and therefore physiologically capable of breeding that season (after the census).

We restricted our models to females, because the vast majority of survival estimates are based on nesting birds (eiders are uniparental incubators; Waltho & Coulson, 2015). Male‐only and aggregated survival estimates were therefore not carried over from the database (*n* = 14 estimates across 8 studies). As females are the limiting sex in ducks, which have male‐biased adult sex ratios, they will generally drive *λ* (Baldassarre & Bolen, 2006). We halved the fertility estimate to account for an approximately equal sex ratio at hatching (Lehikoinen et al., 2008).

Our model partitions ‘pre‐breeder’ into ages of 1 (sexually immature) to 4 years old (final year for recruitment deferral), making use of available age‐stratified recruitment data. Recruitment probabilities were based on (i) estimates of breeding propensity at 2 years old, 3 years old (including repeat breeders) etc.; and (ii) estimates of the proportion of recruits first breeding at each age (which must sum to 1 across all ages). For the purposes of our model, we needed the former, but could improve our estimates by incorporating the latter (see Appendix C).

Intermittent breeding has previously been represented in life cycles through proportionally reduced fertility, including for seaducks (e.g. Flint et al., 2016; Koneff et al., 2017; Tjørnløv et al., 2019). For the common eider, mean adult female breeding propensity has been estimated as 0.72 (Nicol‐Harper et al., 2021b). We incorporated a discrete and reversible ‘nonbreeder’ stage, to which surviving individuals not attempting breeding transition, as determined by breeding propensity: p(nonbreeding) = 1–0.72 = 0.28. Surviving adults can therefore transition: from breeder to breeder (‘continued breeding’) or nonbreeder to breeder, both with probability 0.72, or from nonbreeder to nonbreeder (‘continued non‐breeding’) or breeder to nonbreeder, both with probability 0.28. In the absence of disaggregated survival estimates for nonbreeding and breeding states, we assume these transitions to be underlaid by the same survival probability. Infertility would be accounted for within fertility estimates (e.g. clutch size or hatching success = 0) rather than breeding propensity (infertile females could attempt breeding).

**FIGURE 1:**
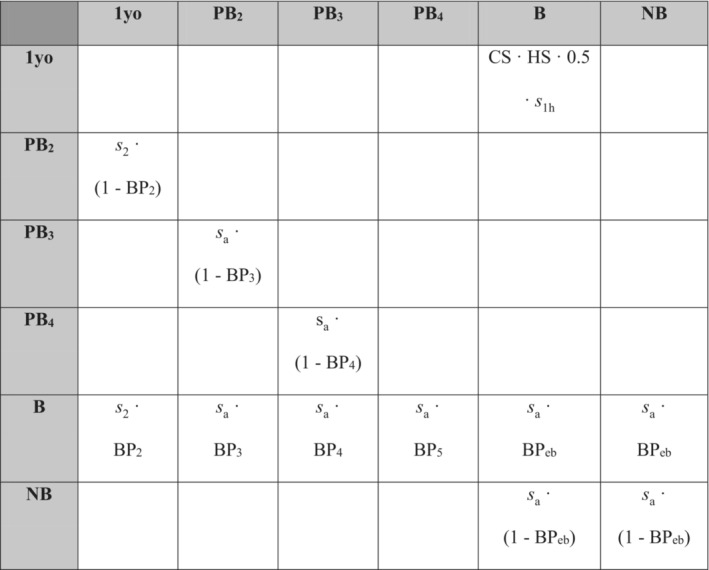
Matrix population model based on our life‐cycle formulation for the common eider. Blank entries represent impossible transitions. 1yo, 1‐year‐old; PB_x_, x‐year‐old prebreeder (e.g. PB_2_, 2‐year‐old prebreeder); B, breeder; NB, nonbreeder; *s*_1h_, first‐year survival from hatching (either measured directly or the product of fledging success and first‐year survival from fledging); *s*_2_, second‐year survival; *s*_a_, adult annual survival; BP_x_, breeding (recruitment) propensity at age x; BP_eb_, breeding propensity of established breeders; CS, clutch size; HS, hatching success. BP_5_ is equal to 1, as all individuals recruit by 5 years old.

In the MPM itself (Figure 1), each matrix entry *a*_*ij*_ represents the contribution of individuals in the *j*th stage (column) of year *t* to the *i*th stage (row) of year *t* + 1. All transitions are subject to survival: individuals alive in year *t* must survive in order to occupy a stage in year *t* + 1. A 1‐year‐old, assuming it survives, transitions either to the breeder stage or to the 2‐year‐old prebreeder stage. An individual surviving to 5 years old must transition to the breeder stage, as this is the oldest observed age of recruitment. Breeders contribute 1‐year‐olds to the following year's population, provided that the eggs laid hatch successfully, the hatchlings fledge successfully, and the fledglings survive until the following year (with the final two transitions being measured either separately or in combination, as *s*_1f_ and FS, or *s*_1h_, respectively). Once an individual has bred, it can transition between breeder and nonbreeder each subsequent year or remain in each stage for any number of years, given survival.

#### 2.2.2 Perturbation analysis and comparison with data collection

We obtained matrix‐element elasticities, the proportional contributions to *λ*, from the elas() function of the R package *popdemo* v1.3–0 (Stott et al., 2018). For comparative purposes, we group elasticities based on transition types: (i) ‘Recruitment’ for transitions to breeding for 1‐ to 4‐year‐olds; (ii) ‘Breeding transitions’ including continued breeding, continued nonbreeding, breeder to nonbreeder, and nonbreeder to breeder; and (iii) ‘Reproduction’ for fertility, that is, breeder to 1‐year‐olds. The corresponding ‘fractional study effort’ on each of these three transition types was calculated as the number of contributing studies for the focal transition type divided by the total number of studies across all vital rates. If all studies consider a given transition type, the ‘fractional study effort’ will be 1 (as in two of the comparative reanalyses mentioned below). Additionally, if studies contribute to more than one of the transition types, the study effort fractions may not sum to one (as for the common eider model here).

For each elasticity, we calculated the deviation of its corresponding fractional study effort from an exact match (i.e. a 1:1 relationship). We then measured the probability of this deviation occurring by chance. For example, an elasticity of 0.3 might have a corresponding fractional study effort of 0.6, which then has a probability of 0.4 (= 1–0.6) of chance over‐representation by at least this; or an elasticity of 0.6 might have a corresponding fractional study effort of 0.3, which then has a probability of 0.3 of chance under‐representation by at least this. We note that this calculation by default appears to penalise any deviation from exact proportionality, whereas in reality the focus would lie on those cases where transitions have been studied far out of proportion with their demographic importance as measured by perturbation analysis.

We further considered the generality of our results through comparative reanalyses, applying our elasticity‐study effort comparison workflow to published demographic meta‐analyses for amphibians (Western toad, *Bufo boreas*, and long‐toed salamander, *Ambystoma macrodactylum*; Vonesh & de la Cruz, 2002), spotted owl (*Strix occidentalis*; Boyce et al., 2005) and black bear (*Ursus americanus*; Beston, 2011). In each case, elasticities were calculated or extracted and compared with the fractional study effort of the relevant vital rates/matrix transitions. For details, see Appendix D and associated R code.

#### 2.2.3 Software and data

Data handling and analysis was implemented in R software v4.0.3 (R Core Team, 2020). Packages were used to handle data (*tidyr* v1.1.4, Wickham, 2021) and generate figures (*metafor* v2.4–0, Viechtbauer, 2010; *RColorBrewer* v1.1.2, Neuwirth, 2014; *forestplot* v1.10.1, Gordon & Lumley, 2020; *fields* v.13.3, Nychka et al., 2021). The underlying database is available from the Dryad Digital Repository [https://doi.org/10.5061/dryad.x3ffbg7ks] (data paper: Nicol‐Harper et al., 2021a); R code and input files for this study are deposited on Figshare (see *Data availability statement*).

## 3 RESULTS

### 3.1 Data availability

Of the 134 studies in the database, 129 were used here. The five unused studies are flagged in the original database as not contributing any vital rates (rather, acting as verified sources for unverified estimates). The numbers of studies and estimates varied greatly among the parameters, with some parameters having multiple estimates per study across years or locations (Table 1). Of the seven studies estimating breeding propensity at 2 years old, a subset of six also estimated breeding propensity at 3, of which two also estimated breeding propensity at 4 and 5. Clutch size, hatch success, fledging success and first‐year survival (from hatching or fledging) all contributed to fertility estimates, with 103 unique studies between them.

**TABLE 1:** Number of studies and independent estimates per vital rate across our database, and estimated mean values as used in our analysis.

Vital rate	Number of studies	Number of independent estimates informing estimated mean	Estimated mean	Variance	Calculation method
*s*_1h_	3	3	0.37	0.08	Simple mean
*s*_1f_	3	3	0.75	0.02	Simple mean
*s*_2_	7	7	0.87	0.008	Simple mean
*s*_a_	35	15	0.86	0.0003	Meta‐analysis
FB	7	7	*x* = 2: 0.17	N/A due to underlying calculations	As described in 2.2.1
			*x* = 3: 0.58		
			*x* = 4: 0.71		
			*x* = 5: 1		
Bl_eb_	7	6	0.72	0.03	Simple mean
CS	91	66	4.08	0.004	Meta‐analysis
HS	27	11	0.61	0.005	Meta‐analysis
FS	13	15	0.22	0.01	Simple mean

*Note*: For the meta‐analysed vital rates (*s*_a_, CS and HS), the number of independent estimates informing the estimated mean refers to the number of estimates used in the meta‐analysis, which excludes estimates without sample sizes, and combines some estimates through sub‐meta‐analysis. Abbreviations as for Figure 1; FB, first breeding (i.e. recruitment propensity at age *x*), FS, fledging success. Estimated means rounded to two decimal places; variances rounded to one significant figure.

### 3.2 Mean vital rate estimates

**FIGURE E1:**
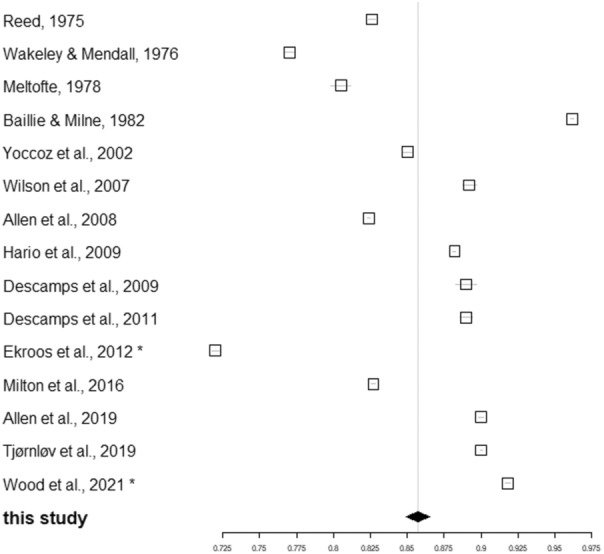
Forest plot for the meta‐analysis of adult survival. Squares show the estimate associated with each study, with size proportional to weighting; whiskers show the 95% confidence intervals around these estimates, based on associated sample sizes and overall mean variance. The diamond and vertical line show the weighted mean resulting from the meta‐analysis. An asterisk denotes mean estimates based on sub‐meta‐analyses.

**FIGURE E2:**
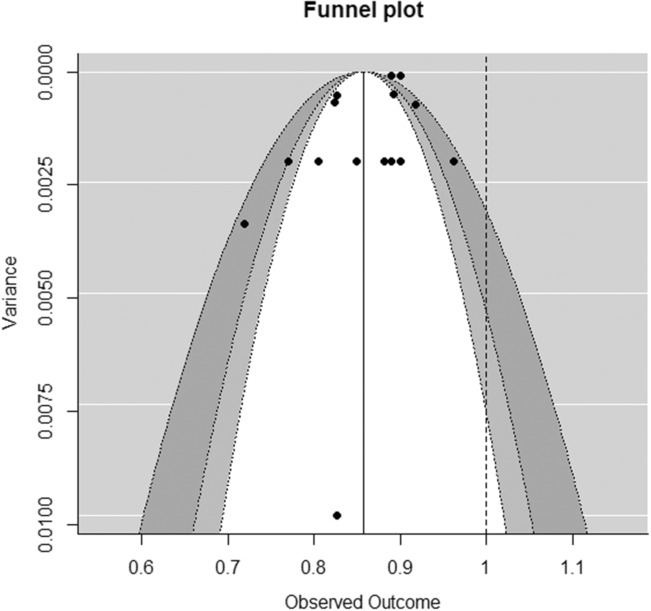
Funnel plot for the meta‐analysis of adult survival, with each estimate (observed outcome) represented by a dot. The solid line shows the overall weighted mean, and the dashed line indicates 1. The white area bounded by a dotted line represents the 90% confidence interval; light grey 95%; and dark grey 99%.

**FIGURE E3:**
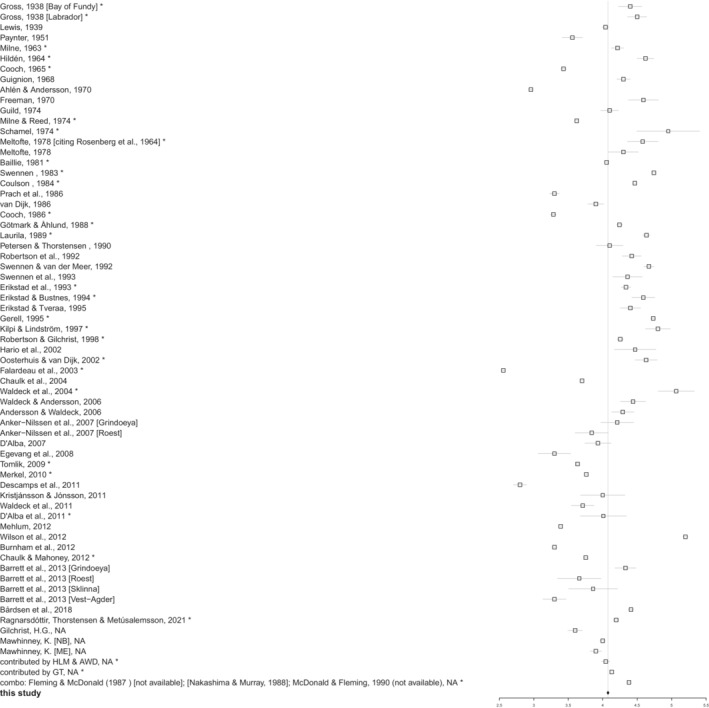
Forest plot for the meta‐analysis of clutch size. Details as for Figure E1.

**FIGURE E4:**
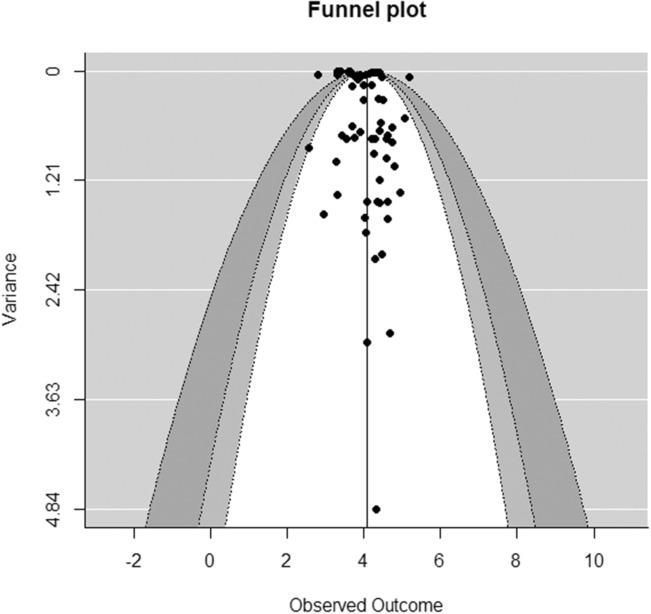
Funnel plot for the meta‐analysis of clutch size. Details as for Figure E2, without the vertical line for 1 as clutch size is not a probability.

**FIGURE E5:**
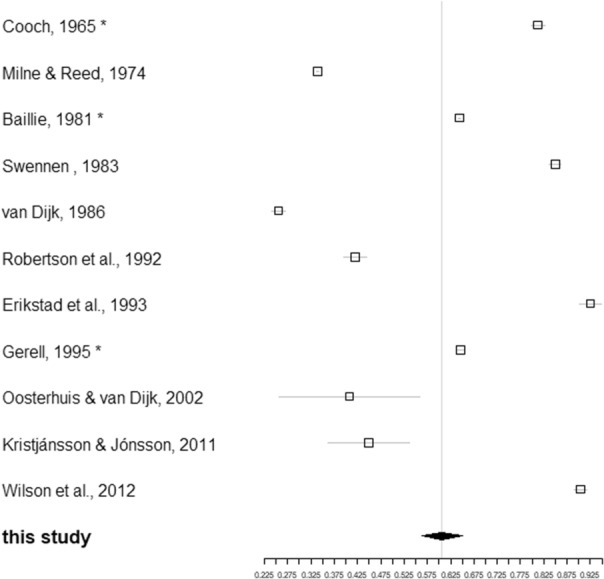
Forest plot for the meta‐analysis of hatching success. Details as for Figure E1.

**FIGURE E6:**
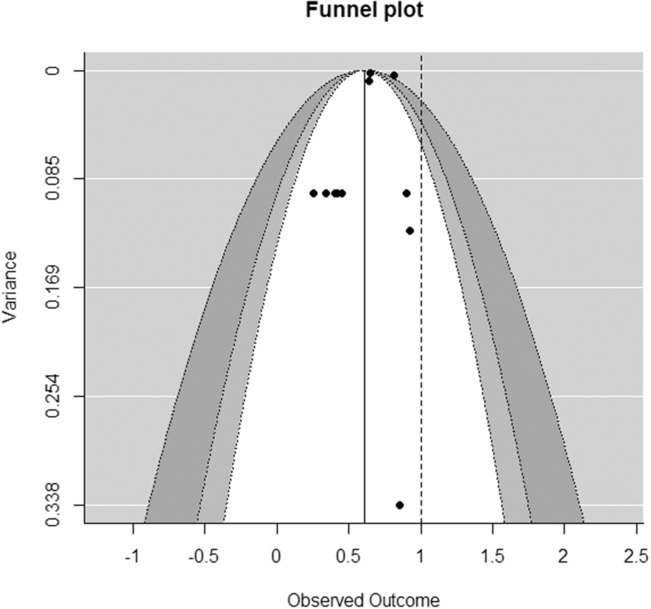
Funnel plot for the meta‐analysis of hatching success. Details as for Figure E2.

We had sufficient estimates (and associated sample sizes) to calculate weighted means for adult survival, clutch size and hatching success; the other vital rates are estimated with a simple mean only (Table 1). The mean estimates for second‐year and adult survival were very similar, and while few individuals recruit at the earliest possible age of 2 years old, by 4 years old breeding propensity is very similar to that of recruited individuals. Forest plots and funnel plots summarising the meta‐analyses are given in Appendix E (Figures E1, E2, E3, E4, E5, E6). While the meta‐analysis is conducted at the species level to facilitate the creation of an overarching population model for the common eider as a whole, mean vital rates across subspecies are provided in Appendix F (Table F1) to allow parameterisation at that level where required.

### 3.3 Parameterised life cycle

**FIGURE 2:**
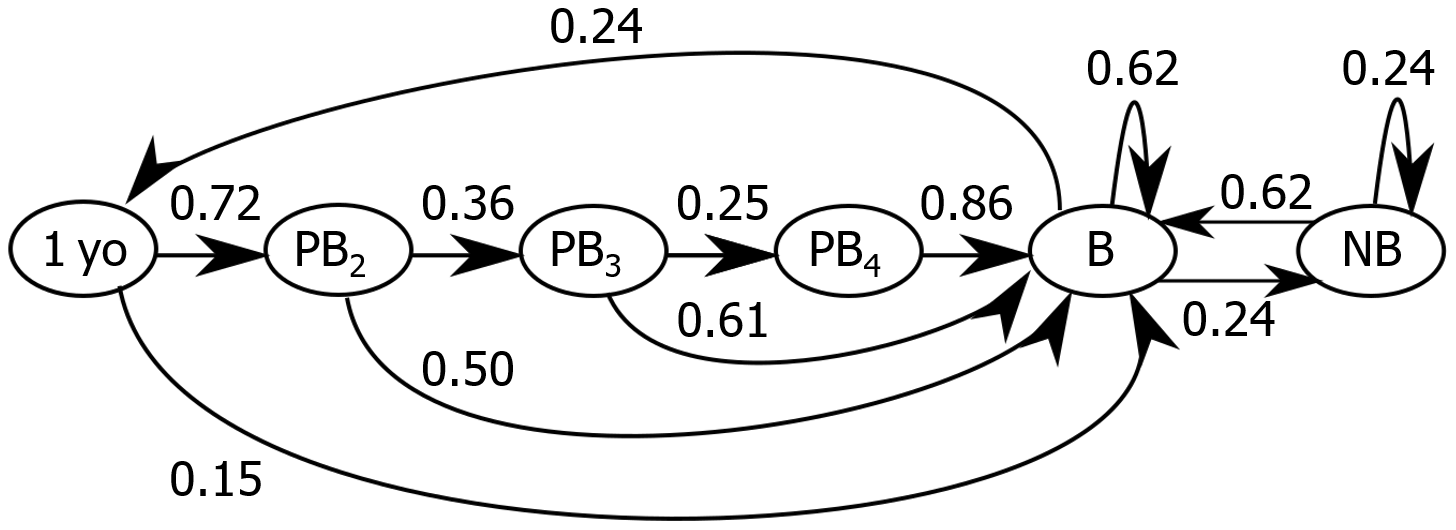
Parameterised life cycle for the common eider, as used in our analysis. Arrows show life‐stage transitions, with stage transition probabilities displayed to two decimal places. Abbreviations as for Figure 1.

The parameterised life cycle shows transitions between stages as calculated from the mean vital rate estimates (Figure 2). The associated *λ* was 0.99 (to two decimal places), representing an expected 1% decline per year for a population at the assumed stable stage structure.

### 3.4 Elasticities

**FIGURE 3:**
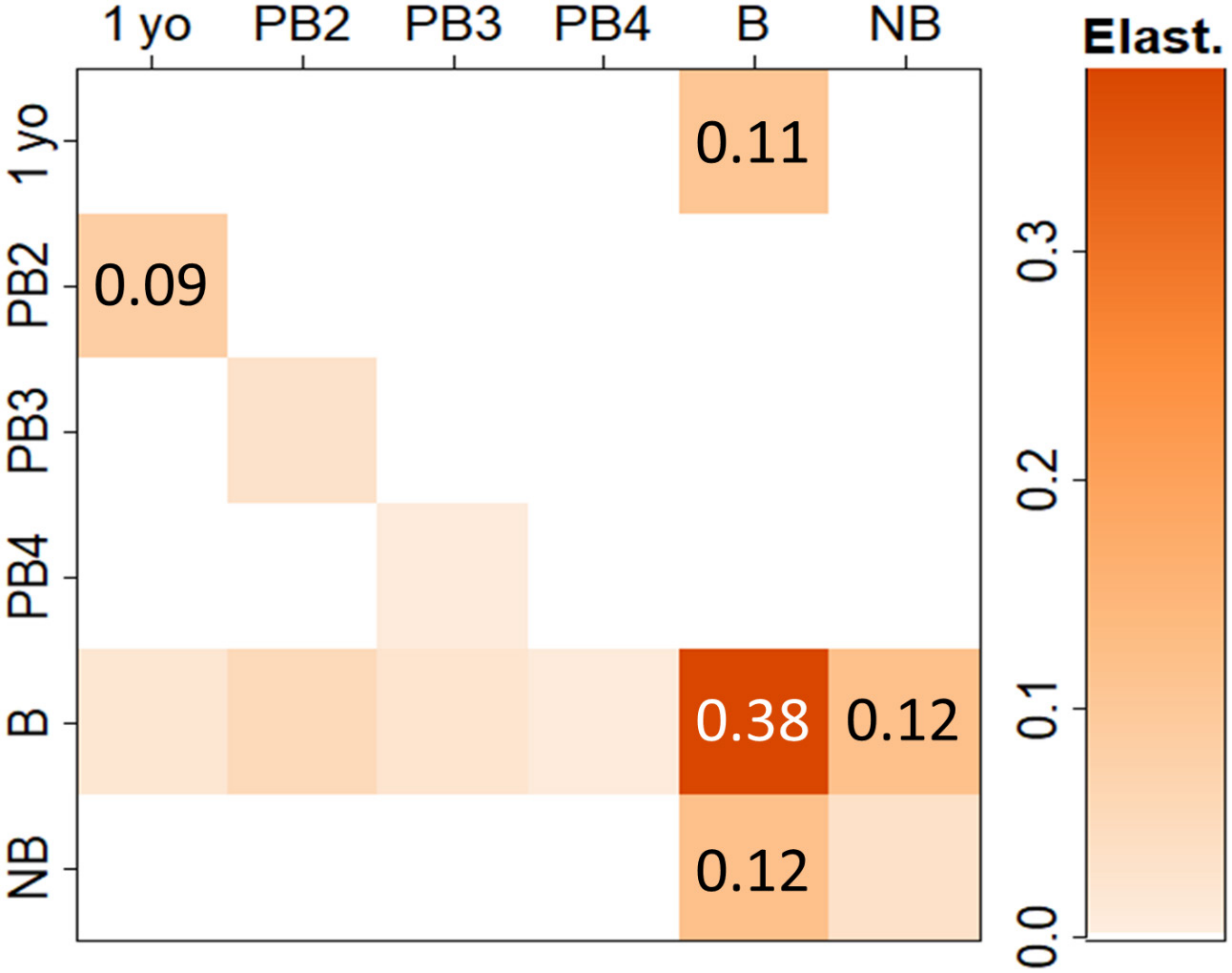
Elasticity matrix, with matrix‐element elasticities shown to two decimal places where >0.05. Abbreviations as for Figure 1; Elast., elasticity. Presentation based on code developed by Steve Ellner and Dylan Childs, available at https://github.com/ipmbook/first‐edition/blob/master/Rcode/utilities/MatrixImage.R.

Transitions between breeding and nonbreeding states (elasticities summing to 24%) had an influence on *λ* that was secondary only to continued breeding (38%) and a much greater influence than fertility (11%, Figure 3). As these elasticities represent the relative influence of the contributing vital rates on *λ*, the importance of directing focus on the transitions between breeding and nonbreeding states is clear.

### 3.5 Comparison of data availability and elasticities

**FIGURE 4:**
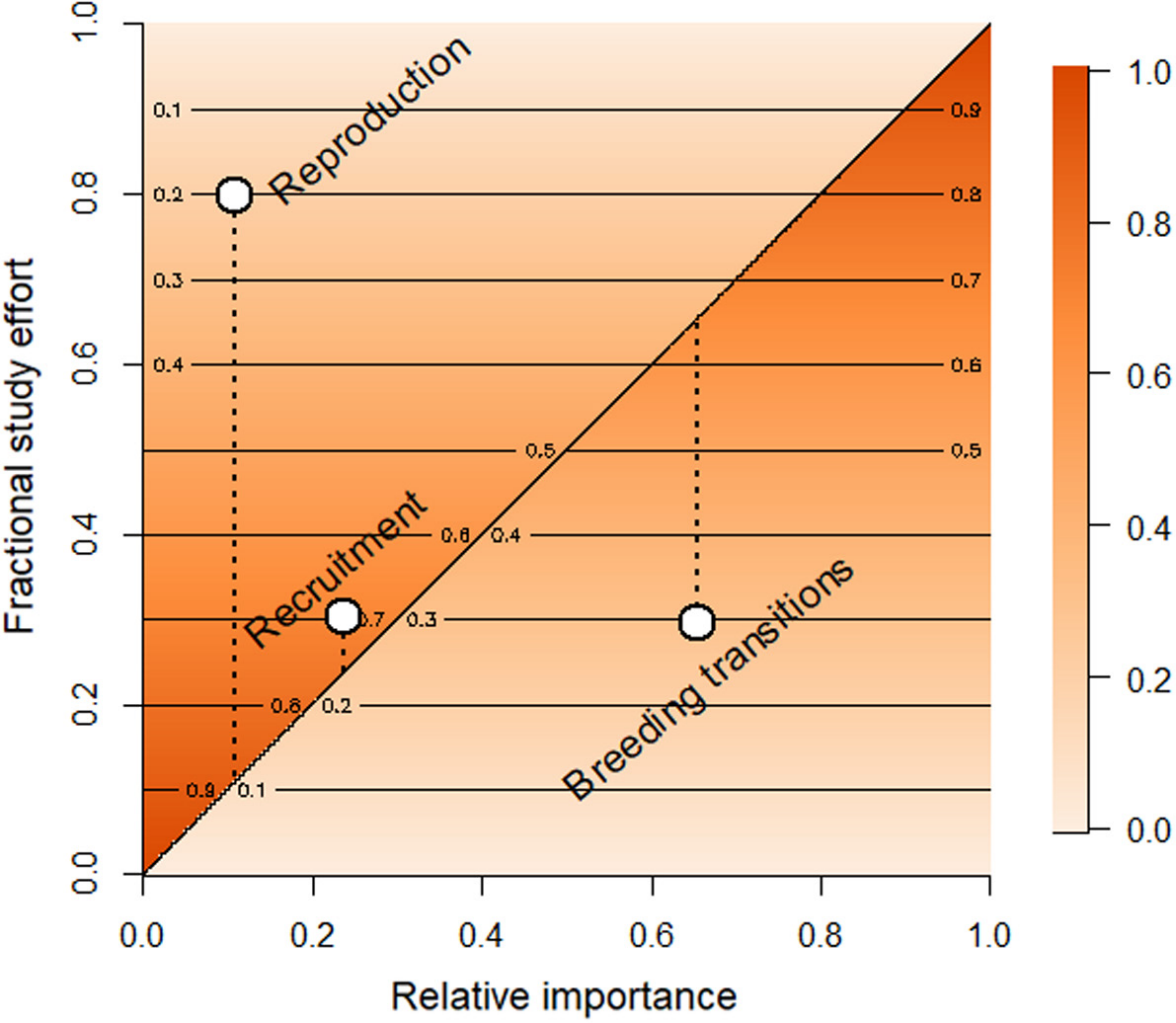
Cross‐plot illustrating fractional study effort against relative importance as measured by matrix‐element elasticities, across grouped common eider life‐cycle transitions. For study effort numerators, we counted 39 ‘Recruitment’ studies, 38 ‘Breeding transition’ studies and 103 ‘Reproduction’ studies; in each case the denominator counts the total of 129 studies across all vital rates. The 1:1 diagonal represents the proportion of studies that would cover each life‐history transition if proportional to importance. Shading and contours represent the probability of a random set having at least as great a deviation from the 1:1 line at each level of importance. For example, a very low relative importance has a high probability of being overrepresented (falling above the 1:1 diagonal), and a high relative importance has a high probability of being under‐represented (falling below the 1:1 diagonal).

We compared the fractional study effort for each grouped transition with their respective contributions to *λ* (summed matrix‐element elasticities). The grouped transitions can be matched to the matrix‐element elasticities in Figure 3 as follows: ‘Reproduction’ represents fertility (in the top row), ‘Breeding transitions’ represent the four transitions in the bottom‐right, and ‘Recruitment’ represents the remaining transitions (i.e. those left of the breeder column). The paired fractions in Figure 4 show that: recruitment has been studied approximately in proportion to its importance in predicting population dynamics; reproduction is overrepresented (largely due to clutch size; see Table 1); and breeding transitions are under‐represented.

This case study of the common eider uncovers a mismatch for this species specifically, but probability analysis shows that disparities will still tend to occur even with more equally distributed elasticities (contours in Figure 4). This is because a random distribution of fractions summing to 1 (e.g. matrix‐element elasticities) more frequently has one high fraction, forcing the others low, than it has one low and several high, and it cannot have several very high fractions. In consequence, disproportionately high data collection will likely be associated with vital rates of low relative importance for the population parameter of interest unless purposefully designed to address this imbalance. Hence, it is unlikely by chance alone that gathering data on the most easily estimated vital rates will cover those vital rates with highest demographic importance.

**FIGURE D1:**
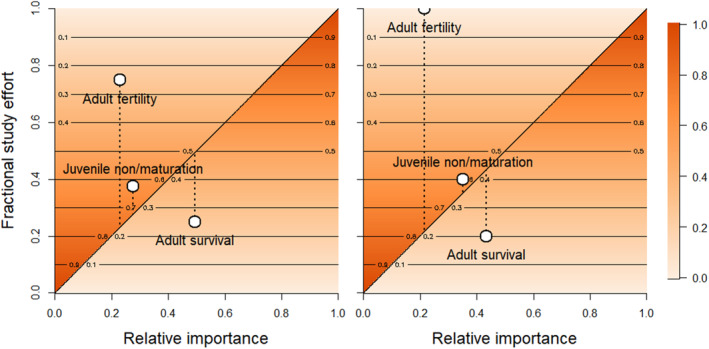
Cross‐plot illustrating fractional study effort against relative importance as measured by matrix‐element elasticities (as for Figure 4 in the main text), across amphibian life‐cycle transitions, for Western toad (left) and long‐toed salamander (right). For fractional study effort numerators, see ‘Number of studies’ in Table D1; for denominators, that is, total studies: Western toad *n* = 8, long‐toed salamander *n* = 5.

**FIGURE D2:**
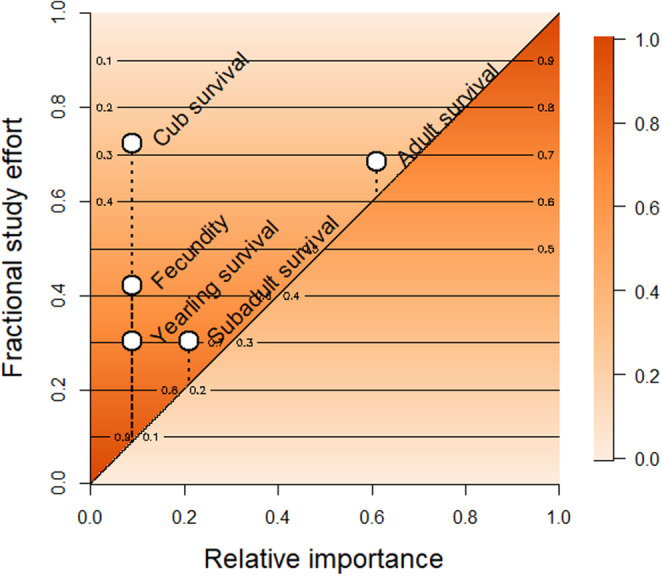
Cross‐plot illustrating fractional study effort against relative importance as measured by matrix‐element elasticities (as for Figure 4 in the main text), across black bear life‐cycle transitions. See Table D3 for study effort values.

Our comparative reanalyses covered a range of possible mismatch scenarios. The two species of amphibian generated similar plots to the common eider, with adult survival relatively understudied and fertility relatively overstudied (Appendix D: Figure D1). The spotted owl represented the special case where (almost) all studies contribute to all transitions, with the study being based on a research programme where each of the three vital rates of interest was recorded at almost all of the sites considered. For the black bear, the elasticity‐‘study effort’ combinations generated a plot where all transitions appear overstudied, although once again early‐life vital rates are overrepresented relative to adult survival (Appendix D: Figure D2).

## 4 DISCUSSION

We have used data synthesis and matrix population modelling to reveal a mismatch across life‐stage transitions between data availability and potential influence on population dynamics across multiple life histories. Specifically, for the common eider, transitions between breeding and nonbreeding are strongly influential on *λ*, and their component vital rates are understudied relative to fertilities. The consequences of such mismatches will depend on the extent of spatiotemporal (co)variation among rates; where there is negligible variation through space and/or time, lower proportional study effort may prove sufficient. On the contrary, any mismatch in rates like breeding propensity that exhibit substantial variation and have a relatively large elasticity would suggest that further data collection would reduce uncertainty in future population projections.

Our meta‐analytic life cycle represents a mean model. Deriving specific parameterisations for given populations could reduce uncertainty in those situations, along with more practical inclusion of temporal (co)variability in vital rates to increase the accuracy of population projections (e.g. see Descamps et al., 2009, Iles, 2012 for examples with the common eider, and Fay et al., 2022 for a demonstration of the importance of estimating vital rate covariance where data availability permits). We do not imply that all vital rates should be studied in direct proportion to the elasticities from prospective perturbation analyses, since such conclusions ‘could be misleading under conditions of either high variance or high uncertainty in vital rates’ (Wisdom et al., 2000, p. 637).

The abundance of data on reproduction, and particularly clutch size, is unsurprising for common eider. Nesting females are colonial, site‐faithful, constant incubators and amenable to close observation, even tolerating being lifted off the nest by hand in some cases (Afton & Paulus, 1992; Waltho & Coulson, 2015). The common eider is a charismatic species, whose reproduction is monitored by researchers, subsistence egg collectors and commercial eiderdown farmers (Cooch, 1986; Jónsson et al., 2009). The number of eggs per nest is straightforward to record on a single visit, although nesting‐season phenology may need to be considered to account for incomplete and partially predated clutches (Paynter, 1951; Sénéchal et al., 2011).

In contrast to fertility, transitions between breeding and nonbreeding states are determined by survival and breeding propensity, which can only be estimated with in‐depth studies involving multiple visits, resighting and/or recovery of individuals. Our mean estimate of adult survival seems fairly low compared with other comparative analyses (Koneff et al., 2017; Waltho & Coulson, 2015); this may be at least partially due to the fact that many estimates relate to apparent rather than true survival and therefore do not account for the possibility of emigration.

Assessments of the role that demographic parameters such as breeding propensity play in population dynamics and hence viability are contingent upon having robust, unbiased datasets. Any uncertainties in the identity of each breeding female in any given year will confound estimates of breeding propensity in ways that cannot readily be captured by confidence intervals. For example, an individual that reappears after a year of absence may have skipped breeding or may have bred without being recorded in nest surveys (Coulson, 2010). A potential solution is the use of dataloggers or telemetry devices. For example, if a breeding female were equipped with a datalogger in year *t*, but not found in year *t* + 1, then subsequent retrieval of the datalogger could suggest incubation in year *t* + 1 with a detected a sustained decrease in light levels, or nonbreeding if the season was spent primarily on water (C. Mitchell, *pers. comms*., 2019). Similarly, the activity patterns of individuals fitted with telemetry devices can indicate whether or not a breeding attempt was made (Weegman et al., 2017).

Our mean vital rate estimates, and therefore our parameterised life cycle, can represent only what is presented in the database. We suspect that many studies assume a breeding propensity of 1 by default (e.g. Kats, 2007). While this could approximate reality for some subpopulations, if breeding propensity has not been measured, then we have no way of assessing that assumption. It follows that untested assumptions of consecutive breeding may mask significantly lower population growth rates. Additionally, *λ* refers to steady state dynamics, whereas the populations providing vital rate and population trend estimates are likely subject to transient effects due to disturbance.

The results of population modelling do not form an end point, but rather part of an iterative process whereby subsequent directed data collection efforts can feed back into improved models (especially where simpler constructions facilitate such flexibility; Ezard et al., 2010). Our contribution here seeks to take the first step down that path: identifying meta‐analytic mean vital rates across the life cycle and then examining proportional study effort to ascertain which life‐stage transitions likely have the most representative coverage across the vast spatiotemporal range that the common eider inhabits.

### 4.1 Wider implications

Our comparative reanalyses of published vital rate meta‐analyses provide evidence for mismatches in other vertebrate species in addition to the common eider, with two species of amphibian (Western toad and long‐toed salamander) similarly exhibiting a relative understudy of adult survival and a relative overstudy of adult fertility. In the black bear study, the specific finding that spatial variation in fecundity appears to drive differences in population growth rates across the range would suggest that this may be the most ‘influential’ vital rate despite a lower elasticity (although the author calls for future research on a range of topics including harvest mortality). In general, in these cases where all transitions appear to be overstudied, the management recommendation might be for studies that are not able to estimate all parameters to prioritise transitions with smallest deviances from the 1:1 line at the expense of those with larger deviances. The comparative reanalyses also demonstrate how our workflow can be replicated in other systems, allowing a more effective use of limited resources to build the most instructive evidence base. Without strategies considering effort distributions where studies cannot estimate all vital rates, data gatherers will tend to overstudy the parameters that contribute least and understudy those that matter most.

In many cases, a principal driver of such mismatches may be the relative ease of collecting data from certain life stages rather than those of most immediate relevance to conservation interventions, as with the accessible hatchlings versus elusive juvenile loggerhead turtles (Crouse et al., 1987). We highlight breeding propensity as a vital rate that is often completely overlooked, even by those attempting to focus on the most important variables. Of the seven studies estimating breeding propensity in the common eider, four studies estimate at least two (and in one case, seven) further vital rates, demonstrating that breeding propensity is not necessarily an overly ambitious addition to existing data‐gathering programmes focussing on other vital rates. More generally, we can still make informed judgements about what is *likely* to be most important in species for which we have little or no demographic data (Conde et al., 2019), by considering better‐studied proxy species and using perturbation analyses as ‘a useful first step in a larger modelling effort to determine population viability’, for example, under environmental stochasticity (Heppell et al., 2000, p. 654).

In contrast, well‐studied rates provide the opportunity to investigate the role of inter‐population variation across geographic ranges and different environments (Frederiksen et al., 2005). Here, we conduct a species‐wide parameterisation with the deliberate aim of presenting an unbiased, universally applicable model for nonlocalised theoretical analyses (as opposed to representing the accurate ‘truth’ for any given subpopulation). Where the focus is on intraspecific variation, the workflow used here could be adapted to those ends. To use the common eider as a specific example, the subspecies means provided in Appendix F could be used to customise our species‐level life‐cycle formulation, or to inform an existing subspecies‐specific version lacking information for a given vital rate or life‐cycle transition. Global‐level data aggregations will necessarily average important subspecific variation.

We are conscious that the available data here are less than all the collected data. There will be other unpublished datasets, as well as data published in languages and sources inaccessible for this study. We do not suggest that data gatherers should stop collecting more easily recorded data such as egg counts, because more data improve precision and accuracy of estimates, and facilitate estimation of regional variation. Furthermore, in some cases data collection may need to be directed towards those vital rates which are most (co)variable, in order to better parameterise the envelope of expected values. For the common eider specifically, Wilson et al. (2012) conclude that while prospective analyses identify adult survival as being highly influential, retrospective analyses highlight past variability in reproduction as a more tractable target for intervention; note that in this study breeding propensity is built into the reproduction term.

We acknowledge that vital rates are usually estimated as part of data‐gathering exercises to answer specific study questions, hence not necessarily with the full life cycle in mind. Conservation policymakers and practitioners are often making best use of the data available to them, which was often collected opportunistically as part of other activities (Dobson et al., 2020). Nevertheless, if our aim is to reduce uncertainty in population projections, then the available data on common eider and other example life histories have a suboptimal distribution across parts of the life cycle. A ‘the‐more‐the‐better’ maxim does not negate the fact that not all data are equally useful; in some cases, the most useful data will be those necessitating a redirection of effort from vital rates that are easier to measure but less informative, in favour of more targeted application. Canessa et al. (2015) demonstrate how ‘value of information’ analysis can be used to determine the expected benefits of investment in obtaining further information, including in a demographic context.

While return on investment should be at the core of each funding body's ethos, conservation is not a top‐down enterprise; strategic decisions, and funding thereof, tend to be made at the level of individual organisations, rather than across all agencies collectively managing data gathering for a particular species. Nevertheless, there are examples of a more strategic approach: of particular relevance to the common eider is the Sea Duck Joint Venture, which publicised a ‘strategic shift in focus’ towards a programme ‘intended to provide information most needed by managers to make informed decisions’ (SDJV Management Board, 2014, p. 4). For the common eider, monitoring and management priorities have been identified at a regional level for the *dresseri* subspecies through elicitation of expert opinion across a coalition of researchers and practitioners (Noel et al., 2021). More broadly, multinational species conservation action plans provide a means of highlighting the relative value of demographic data to researchers, which could help to direct research efforts towards the data most needed to inform conservation assessments. A relevant example would be the International Single Species Action Plan for some populations of the common eider produced by the African‐Eurasian Waterbird Agreement (Lehikoinen et al., 2022). More generally, the British Trust for Ornithology is already very successful in directly enthusing citizen scientists to collect specific types of data.

Strategic projects nevertheless require long‐term resourcing to ensure sustainable collection and maintenance of the valuable individual‐based longitudinal datasets required to parameterise full life cycles of long‐lived species (Culina et al., 2020). The focus here is on long‐term population growth rate as the conservation target, but such datasets support a range of outputs, not least the vital rate estimates themselves. Additionally, consideration of MPM‐derived priorities may necessitate greater engagement by population ecologists, in demonstrating the utility of population models and exactly what data are required for them. Green (1995) and Frederiksen et al. (2014) highlight the fundamental role of population models in species recovery and make a strong case for collaboration between biologists modelling population declines and conservationists making action plans to reverse them. To this end, we hope that our workflow with its use of relatively simple MPMs may help to reduce barriers to uptake.

In conclusion, our results highlight a propensity for disconnects between empirical demographic data and the information needs of conservation biologists and wildlife managers. The motivations for on‐the‐ground conservation efforts may be different from what is needed to reduce uncertainty in population projections, but we need to start by counting what we have, to work out how we can iteratively improve evidence‐led conservation. Where perturbation analysis is used to inform investments into future conservation research and interventions—ideally in combination with considerations of transient dynamics, stochasticity, uncertainty, (co)variability and tractability—alignment between data collection and demographic influence where practicable would be valuable, particularly where vital rates are both variable and influential on population growth. Enhanced collaboration and co‐ordination between citizen scientists, ecologists and population modellers to co‐produce greater knowledge around the difficult‐to‐measure rates would help manage taxa of conservation concern most effectively.

## AUTHOR CONTRIBUTIONS

**Alex Nicol‐Harper:** Conceptualization (equal); data curation (lead); formal analysis (lead); methodology (equal); software (lead); writing – original draft (lead); writing – review and editing (equal). **C. Patrick Doncaster:** Methodology (equal); supervision (equal); writing – review and editing (equal). **Geoff M. Hilton:** Supervision (equal); writing – review and editing (equal). **Kevin A. Wood:** Supervision (equal); writing – review and editing (equal). **Thomas H. G. Ezard:** Conceptualization (equal); supervision (equal); writing – review and editing (equal).

## CONFLICT OF INTEREST STATEMENT

The authors declare no competing interests.

## Figures and Tables

**FIGURE B1:**
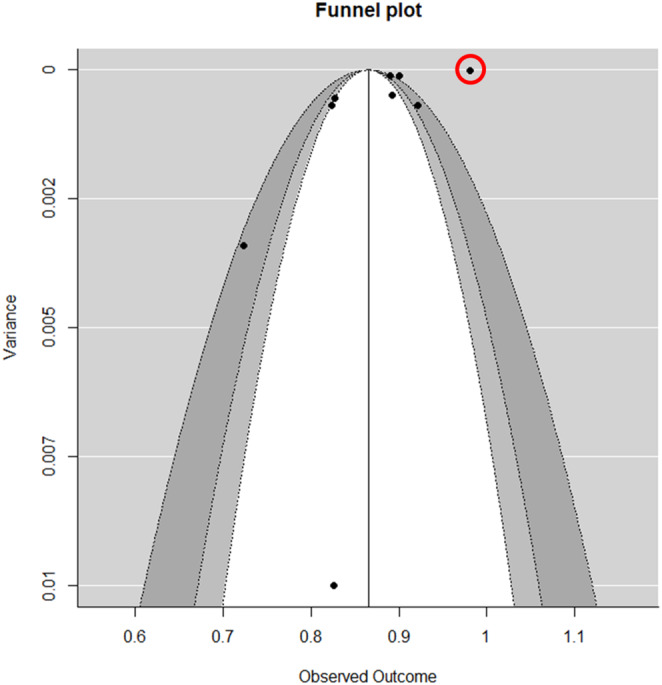
Funnel plot for adult survival estimates, following sub‐meta‐analysis, removal of estimates without associated sample sizes and removal of one outlier (estimate = 0.51). The remaining outlier, well outside the bounds of the 99% confidence interval, is circled in red.

## Data Availability

Underlying data and code are available on Figshare: input files: https://doi.org/10.6084/m9.figshare.16832686; output code: https://doi.org/10.6084/m9.figshare.16832878; comparative code: https://doi.org/10.6084/m9.figshare.16832884.

## APPENDIX A SENSITIVITY ANALYSIS ON INCLUSION OF UNVERIFIED ESTIMATES AND ESTIMATES BASED ON RANGE MIDPOINTS

We analysed the sensitivity of vital rate estimate means to inclusion of unverified estimates, and those based on range midpoints, using Wilcoxon rank‐sum tests with continuity correction (Table A1). These are the nonparametric version of t‐tests, since visual inspection of histograms, and *p*‐values <.05 in Shapiro–Wilk tests, suggested rejection of normal distributions for the estimates of each vital rate (Table A1). The tests were run on the full dataset as taken from the database, that is, including nonindependent estimates, and prior to sub‐meta‐analyses. The Wilcoxon tests compared the full dataset to a subset excluding unverified or midpoint estimates, respectively.

Shapiro–Wilk test: *p* < .05 → not normally distributed.

Wilcoxon test: *p* > .05 → not significantly different.

**TABLE A1:** Outputs from Shapiro–Wilk and Wilcoxon tests on adult survival, clutch size and hatching success.

Vital rate	Shapiro–Wilk test	Wilcoxon test—Unverified estimates	Wilcoxon test—Range midpoints
Adult survival	*W* = 0.81176	*W* = 1345.5	*W* = 1599.5
	*p*‐value = .0000004563	*p*‐value = .7488	*p*‐value = .8544
Clutch size	*W* = 0.97845	*W* = 35,161	*W* = 42,091
	*p*‐value = .0002124	*p*‐value = .9346	*p*‐value = .9244
Hatching success	*W* = 0.95294	*W* = 1261.5	*W* = 1390
	*p*‐value = .03107	*p*‐value = .8706	*p*‐value = .8055

## APPENDIX B EXAMPLE META‐ANALYSIS

Here, we work through the meta‐analysis process for adult survival, starting with the sub‐meta‐analysis conducted on two studies and then describing the overall meta‐analysis process for the resulting set of comparable estimates. See Section 2.1.2 for further detail and citations regarding justification of these methods.

For adult survival, two studies provided multiple estimates requiring sub‐meta‐analysis: ID's 90 (Wood et al., 2021) and 91 (Ekroos et al., 2012). A simplified extract from the input file (based on the vital rate database in Nicol‐Harper et al., 2021b) shows that they both provide multiple estimates, standard errors and sample sizes, across colonies and island cover types, respectively (Table B1). Note that selecting, for example, a single estimate per study based on maximal precision (i.e. lowest provided standard error) or replication (i.e. largest sample size) would send forward to the main meta‐analysis higher estimates than taking a mean (whether weighted or unweighted).

**TABLE B1:** Simplified extract from the adult survival input file, showing studies to undergo sub‐meta‐analysis.

ID	Estimate	SE.prov	*n*	Comments	rm_ind. justification
90	0.94	0.015	1192	Akureyri	For weighted mean across different colonies
90	0.887	0.027	821	Flatey	For weighted mean across different colonies
90	0.922	0.017	1005	Rif	For weighted mean across different colonies
91	0.679	0.027	566	Forested	For weighted mean across island types
91	0.761	0.013	566	Open	For weighted mean across island types

*Note*: SE.prov = provided standard error; *n* = sample size; rm_ind.justification explains why the estimates for each study should undergo sub‐meta‐analysis.

Here, we show the calculations involved in the sub‐meta‐analysis for Wood et al. (2021):

Step 1. Convert standard errors to variances [variance = (SE × √sample size)^2^]:

(0.015 × √1192)^2^ = 0.268200.
(0.027 × √821)^2^ = 0.598509.
(0.017 × √1005)^2^ = 0.290445.

This step would be skipped if variances were provided by the studies, or if standard errors based on survival estimates from mark–recapture modelling had already been converted to variances as described in Section 2.1.2.

Step 2. Calculate mean variance:

(0.268200 + 0.598509 + 0.290445)/3 = 0.385718.

Step 3. Calculate study‐level error variances, *v*_*i*_'s, as shown in main text Section 2.1.2:

0.385718/1192 = 0.0003235889.
0.385718/821 = 0.0004698149.
0.385718/1005 = 0.000383799.

Step 4. Calculate weightings, *W*_*i*_'s, as shown in main text Section 2.1.2:

Var (0.94, 0.887, 0.922) = 0.0007263333.
Mean (0.0003235889, 0.0004698149, 0.000383799) = 0.0003924009.
*T* ^2^ = 0.0007263333–0.0003924009 = 0.0003339324.
1/(0.0003235889 + 0.0003339324) = 1520.863.
1/(0.0004698149 + 0.0003339324) = 1244.172.
1/(0.000383799 + 0.0003339324) = 1393.279.

Step 5. Calculate the meta‐estimate and meta‐variance, as shown in main text Section 2.1.2:

1520.863 + 1244.172 + 1393.279 = 4158.314.
(1520.863 × 0.94 + 1244.172 × 0.887 + 1393.279 × 0.922)/4158.314 = 0.9181113.
1/4158.314 = 0.0002404821.

Step 6. Meta‐variance is then multiplied by the number of contributing estimates, for compatibility with calculated variance from studies without sub‐meta‐analysis within the main meta‐analysis:

0.0002228807 × 3 = 0.0007214463.

That is, across the three estimates [0.887, 0.922, 0.94] in Wood et al. (2021), the weighted mean adult survival is 0.918 (to three significant figures) with a meta‐variance of 0.0002 and associated study‐level variance of 0.0007 (both to one significant figure). This is equivalent to 0.922 ± 0.03 SD (to three significant figures). The mean value is higher than when calculating a simple mean (0.916 ± 0.03 SD), because the two higher estimates are associated with greater precision. The equivalent process with the two estimates from Ekroos et al. (2012) generates meta‐estimate = 0.72, meta‐variance = 0.002, study‐level variance = 0.003, SD = 0.06.

These two estimates generated by sub‐meta‐analyses can then be handled equivalently to single estimates from other studies (often representing means where disaggregated data were not provided). Following the removal of estimates without associated sample sizes, the adult survival dataset for meta‐analysis is as shown in Table B2.

**TABLE B2:** Simplified extract from the adult survival dataset, following sub‐meta‐analyses and removal of estimates without associated sample sizes.

ID	Estimate	Var.prov	Var.calc	SD.prov	SE.prov	*n*
2	0.9616	NA	NA	NA	NA	6000
5	0.981	NA	0.000004	NA	0.002	2238
17	0.51	NA	NA	NA	0.0011	1166
38	0.805	NA	NA	NA	NA	163
66	0.882	NA	NA	NA	NA	6393
67	0.77	NA	NA	NA	NA	650
70	0.892	NA	0.000484	NA	0.022	361
71	0.826	NA	NA	0.099	NA	1118
72	0.85	NA	NA	NA	NA	862
74	0.9	NA	0.0001	NA	0.01	2340
76	0.827	NA	NA	0.023	NA	3028
77	0.89	NA	NA	NA	NA	150
92	0.89	NA	0.0001	NA	0.01	398
97	0.9	NA	NA	NA	NA	22,320
120	0.824	NA	0.000676	NA	0.026	6500
90	0.918111	NA	0.000721	NA	NA	3018
91	0.72	NA	0.003362	NA	NA	1132

*Note*: Var.prov = provided variance; Var.calc = calculated variance; SD = standard deviation; other variables as in Table B1. The meta‐variances calculated from the sub‐meta‐analyses have become ‘calculated variances’, which are later further populated by conversion from provided standard deviations/errors from other studies where available. Beforehand, in this case two outliers are removed upon inspection: 0.51 (ID 17) as it is >2 SD below the mean value and 0.981 (ID 5) as it substantially exceeds confidence limit bounds in the funnel plot (Figure B1).

The mean calculated variance across all available values (including converted from SD/SEs) = 0.001971681 and the overall weighted mean adult survival = 0.857 ± 0.0003 variance or 0.02 SD. Sensitivity analysis (code not provided) showed that adult survival would remain 0.857 to three decimal places if simple means rather than weighted means were applied to the ‘sub‐meta‐analysis’ studies, or even if taking a simple mean rather than applying Doncaster and Spake's (2018) methodology across the meta‐analysis dataset, with simple means applied at the study level also. The overall value would, however, be 0.861 if the most precise estimates from Ekroos et al. (2012) and Wood et al. (2021) were sent forward in place of a mean across their replicates.

For the full method, and the equivalent process for clutch size and hatching success, see provided code.

## APPENDIX C RECRUITMENT PROPENSITIES

Recruitment probabilities were based on (i) estimates of breeding propensity at 2 years old, 3 years old (including repeat breeders), etc. and (ii) estimates of proportion of recruits first breeding at each age (which must sum to 1 across all ages). The two types of estimate can be combined through the following equality: the proportion of individuals that first breed at age *x* is equal to the proportion that survive to age *x* (without yet breeding) and then breed at age *x*. Since we have survival rates and do not need to know the proportion of individuals that recruit, we can solve simultaneous equations for the breeding propensity at age *x* for 2 ≤ *x* ≥ 4 (with breeding propensity at 5 years old set to 1, since there is no evidence of any recruitment beyond age 5). The resulting set of simultaneous equations simplifies algebraically to give the age‐specific breeding propensities in terms of survival rates and probabilities of first breeding at age *x*. The validity of these calculations was checked via back‐substitution, ascertaining that the proportion of 1‐year‐olds going on to recruit tallies with the complement of the proportion of individuals dying before breeding (at any of 2, 3, 4 or 5 years old). Calculations available upon request.

## APPENDIX D COMPARATIVE ANALYSIS ACROSS ADDITIONAL PUBLISHED STUDIES

Here, we consider the generality of our results from the common eider case study through comparative reanalyses, applying our elasticity‐‘study effort’ workflow to the published demographic meta‐analyses detailed below (methods adapted from Section 2.2 as detailed for each example). Note that we selected demographic meta‐analyses with associated MPMs to facilitate direct comparison. Outputs can be replicated with our provided R code.

- Vonesh and De la Cruz (2002)—amphibians:

This study considers ‘two amphibians with contrasting life‐history strategies’ (Western toad, *Bufo boreas*, and long‐toed salamander, *Ambystoma macrodactylum*), through application of a ‘generalized amphibian life cycle’ (p. 325). We used ‘Fixed’ estimates from their table 2 to parameterise the provided matrix (their equation 3) for each species (see Table D1). Study effort fractions were based on the references in their table 2; while their study is not strictly a meta‐analysis, most transitions are based on information from multiple studies (see Table D2). Juvenile non/maturation transitions were pooled for the comparison as they are based on the same vital rates and therefore shared data collection.

For both species, adult survival was relatively understudied while adult fertility was relatively overstudied (Figure D1); the study results confirm greater sensitivity to postembryonic survival than egg survival (a component of fertility) across a range of density‐dependence scenarios. For long‐toed salamander, all five studies contributed to adult fertility, so the study effort fraction is 1.

**TABLE D1:** MPMs for (a) Western toad and (b) long‐toed salamander, as derived from equation 3 and table 2 in Vonesh and De la Cruz (2002) and Table D2. These are generated in our provided R script as ‘matrix1’, with a prompt for the user to select the species (‘*n* = 1’ for Western toad or ‘*n* = 2’ for long‐toed salamander). For both species, the matrix transition rates are defined as follows.

[1,1]: juvenile nonmaturation = juvenile survival × (1 – maturation).		
[1,2]: reproduction = clutch size × egg survival × metamorph survival × density‐dependent coefficient × maximum larval survival × sex ratio (assumed equal)		
[2,1]: juvenile maturation = juvenile survival × maturation		
[2,2]: stasis = adult survival		
**(a)**	**Juvenile**	**Adult**
Juvenile	0.150	4.032
Adult	0.050	0.600
[1,1]: 0.2 × (1–0.25); [1,2]: 12000 × 0.6 × 0.2 × 0.007 × 0.8 × 0.5; [2,1]: 0.2 × 0.25.		
**(b)**		
Juvenile	0.348	0.648
Adult	0.252	0.600
[1,1]: 0.6 × (1–0.42); [1,2]: 90 × 0.6 × 0.6 × 0.05 × 0.8 × 0.5; [2,1]: 0.6 × 0.42.		

**TABLE D2:** Summary of information extracted from Vonesh and De la Cruz (2002).

Matrix element/transition	Contributing vital rates	Number of studies
[1,1] juvenile → juvenile (nonmaturation)	Juvenile survival; Maturation probability	WT: 3 LTS: 1
[1,2] adult → juvenile (reproduction)	Clutch size; Egg survival; Metamorph survival; Density‐dependent coefficient; Maximum larval survival; Sex ratio (assumed equal)	WT: 6 LTS: 5
[2,1] juvenile → adult (maturation)	Juvenile survival; Maturation probability	WT: 3 LTS: 1
[2,2] adult → adult (stasis)	Adult survival	WT: 2 LTS: 1

Abbreviations: LTS, long‐toed salamander; WT, Western toad.

- Boyce et al. (2005)—spotted owl:

This study refers to three data summaries (Anderson & Burnham, 1992; Burnham et al., 1996; Franklin, 1992) all covering juvenile survival, adult survival and adult fecundity, with overlapping study areas such that the results are nonindependent (see their tables 2 and 3; e.g. presumably the values for study area CAL in the year 1999 meta‐analysis include some of the contributing studies for the 1996 and/or 1992 analyses). Given that 22 or 23 of the total 23 sites contribute to each vital rate in at least one summary, this essentially represents a case where all three vital rates have been measured in each study; that is, study effort fractions equal to 1 (or 22/23). Hence, there is no need to plot a comparative figure, since all vital rates would appear overstudied, as opposed to having almost equal (near complete) coverage.

- Beston (2011)—black bear:

This study describes a demographic meta‐analysis for black bear across North America. We used the values provided on p. 1591 for fractional study effort (see Figure D2 legend) and their table 5 for vital rate elasticities (taking a simple mean across the two geographic areas); see Table D3. Plotting these directly, all transitions are shown to be overstudied, but especially cub survival and fecundity (Figure D2). In this case, the recommendation might be for studies that are not able to calculate all parameters to prioritise sub/adult survival at the expense of fecundity and cub survival. In this case, the author discusses the fact that ‘larger differences in fecundity (a vital rate with lower sensitivity [and elasticity]) between east and west outweighed smaller differences in survival (a vital rate with higher sensitivity [and elasticity])’ (Beston, 2011, p. 1590). They go on to call for the collection of further data (mark–recapture, movement tracking and harvest assessment) and specific study of the costs of reproductive costs and compensatory mortality.

**TABLE D3:** Summary of information extracted from Beston (2011).

Vital rate	Number of studies; total = 76	Provided elasticities (mean)
Cub survival	55	0.07–0.11 (0.09)
Yearling survival	23	0.07–0.11 (0.09)
Subadult survival	23	0.20–0.22 (0.21)
Adult survival	52	0.55–0.67 (0.61)
Fecundity	32	0.07–0.11 (0.09)

## APPENDIX E META‐ANALYSIS FOREST PLOTS AND FUNNEL PLOTS

For references, see ‘Data sources’ in Nicol‐Harper et al. (2021b).

## APPENDIX F MEAN VITAL RATES ACROSS SUBSPECIES

**TABLE F1:** Mean vital rates across *Somateria mollissima* subspecies. Abbreviations as for Table 1; values are given to two decimal places, and sample size refers to the number of studies. The database contains no estimates for *S.m. faroeensis*.

	*borealis*	*dresseri*	*faroeensis*	*mollissima*	*sedentaria*	*v‐nigrum*
*s*_1h_	0.50 (*n* = 2)	N/A	N/A	0.12 (*n* = 1)	N/A	N/A
*s*_1f_	N/A	0.65 (*n* = 1)	N/A	0.80 (*n* = 2)	N/A	N/A
*s*_2_	0.76 (*n* = 2)	0.89 (*n* = 1)	N/A	0.91 (*n* = 4)	N/A	N/A
*s*_a_	0.89 (*n* = 9)	0.84 (*n* = 6)	N/A	0.86 (*n* = 25)	N/A	0.89 (*n* = 1)
FB	N/A due to underlying calculations (but 16/18 estimates relate to *mollissima*)					
BP_eb_	0.45 (*n* = 1)	0.92 (*n* = 1)	N/A	0.74 (*n* = 4)	N/A	N/A
CS	3.64 (*n* = 47)	3.93 (*n* = 28)	N/A	4.37 (*n* = 140)	4.35 (*n* = 20)	4.81 (*n* = 6)
HS	0.62 (*n* = 7)	0.37 (*n* = 7)	N/A	0.71 (*n* = 25)	0.42 (*n* = 1)	0.73 (*n* = 2)
FS	N/A	0.22 (*n* = 3)	N/A	0.23 (*n* = 11)	N/A	0.19 (*n* = 1)
